# The Pectoral (PECS) Regional Block: A Scoping Review

**DOI:** 10.7759/cureus.46594

**Published:** 2023-10-06

**Authors:** Khalid Bin Ghali, Nourah AlKharraz, Omar Almisnid, Adel Alqarni, Omar A Alyamani

**Affiliations:** 1 Anesthesiology, King Faisal Specialist Hospital and Research Centre, Riyadh, SAU; 2 Anesthesiology, Qassim University, Qassim, SAU; 3 College of Medicine, King Saud University, Riyadh, SAU

**Keywords:** pain management, pectoralis nerve block, anesthesiology, regional anesthesia, pecs block

## Abstract

Among the various surgical procedures, breast surgeries rank as a frequently conducted procedure. Interfacial blocks such as the Pectoral (PECS) block became possible with the currently available knowledge on innervations and ultrasound. Interfacial blocks target the deep fascial planes, which are potential spaces for injecting local anesthetics. The Pectoral I (PECS I) consists of the injection of local anesthetics in the plane between the pectoralis major and minor muscles. The PECS II block, a modified version of the block, is achieved by adding another, deeper injection in the plane between the pectoralis minor and the serratus anterior muscle. We conducted a scoping review using Arkesy and O’Malley's framework, as described by Levac. We identified our research question as the uses of the PECS regional block technique with the choice of local anesthetics, including adjuncts, and its effectiveness in intraoperative and postoperative analgesia in the first 24 hours and incidence of postoperative nausea and vomiting. Subsequently, we identified the relevant studies that met our inclusion criteria and charted the data. Lastly, we summarized and reported the results. The PECS block was used in various breast surgeries, among which radical mastectomies with/without lymph node dissection were the most common. It was found that the PECS block reduced intraoperative opioid consumption in 60% and 24-hour postoperative opioid consumption in 93.3% of the included papers. Various local anesthetics were used such as ropivacaine, bupivacaine, and levobupivacaine. Ultrasound-guided interfacial plane blocks, such as the PECS block, are a recent development in regional anesthesia that offers analgesia for patients undergoing breast surgeries. The authors conclude that PECS block can provide a decrease in intraoperative and postoperative opioid consumption, a decrease in the incidence of nausea and vomiting, and can lead to overall patient satisfaction in terms of lower pain scores compared to systemic analgesia.

## Introduction and background

Breast surgery is one of the most common types of surgery worldwide [1]. These surgeries are accompanied by significant pain and have a high incidence of chronic pain postoperatively, even following minor procedures [2]. Poor management of perioperative pain can lead to chronic pain, psychological trauma, reduced quality of life, as well as delayed functional recovery, and post-anesthesia care unit discharge [3,4]. Regional blocks have been implemented in the perioperative setting to provide superior pain control. Additionally, regional blocks have the potential benefits of decreasing the incidence of chronic pain and reducing opioid consumption and postoperative pulmonary complications. With lower opioid consumption, a lower rate of adverse effects (nausea, vomiting, respiratory complications, hyperalgesia, and immunosuppression) can be achieved [5].^ ^The Pectoral I (PECS I) block was first described by Blanco in 2011. In this technique, local anesthetics are injected in the plane between the major and minor pectoralis muscles to achieve a block of the medial and lateral pectoral nerves. As these nerves mostly innervate the pectoralis muscles, the PECS I block is theoretically suited for surgery involving these muscles. In 2012, the same author proposed a modified version of the block called the PECS II block. It is achieved by adding another, deeper injection in the plane between the pectoralis minor and the serratus anterior muscle. This technique is believed to contribute to more extensive anesthesia of the chest wall by also blocking the long thoracic nerve and the lateral branches of the intercostal nerves from T3 to T6 _ _[6]. We conducted a scoping review to compare the type of block performed for each type of breast surgery, the local anesthetic used, intraoperative opioid use, postoperative pain, postoperative opioid consumption, and postoperative nausea and vomiting.

## Review

Methodology

This study was conducted using Arkesy and O’Malley’s framework for scoping reviews, as described by Levac, consisting of five stages described below [7,8].

Stage 1: Identifying the Research Question

To capture a wide range of publications, we identified broad research questions to identify current practices and their advantages. Identifying the questions was a continual process between the authors which resulted in the following questions: (1) What are the uses of the PECS block regional technique? (2) Is the PECS block regional technique effective for intraoperative and postoperative analgesia in the first 24 hours? (3) What type of local anesthetic and adjuvants were used?

Stage 2: Identifying Relevant Studies

The following search terms were used: PECS block, regional anesthesia, breast surgery, and pectoralis nerve block. Two independent authors searched five databases (MEDLINE, EMBASE, EBSCO, Scopus, and Web of Science) on September 3, 2022. We limited our search to articles published since 2010 to provide an overview of past and current PECS block techniques. Publications were collected using a shared bibliography, and duplicate articles were removed.

Stage 3: Selecting Studies for Inclusion

A total of 22 articles were identified during the first search, and 15 articles were included in our scoping review. The inclusion criteria were original investigations, articles published in English, original data presented in the study, and a description of the use of PECS regional block for patients undergoing breast surgery.

We included studies that involved adult patients undergoing breast surgery with PECS regional block as the primary modality of anesthesia or as part of the postoperative analgesia plan. Studies published in English between 2010 and September 2022 were included in the review. All original articles were included (case reports, case series, clinical trials, and systematic reviews). Studies were excluded if not in the English language and/or if described as an ongoing research protocol.

Stage 4: Charting the Data

For every study, we extracted author names, year of publication, study title, article type, the aim of the study, methodology used, outcomes that were assessed, population, results, anesthesia technique, block technique, drugs that were used, inclusion and exclusion criteria, number of participants, and conclusion.

The appropriate study data was condensed in a tabulated form; two authors performed this step independently for all articles, and a final table was compiled after a discussion between the authors.

Stage 5: Summarizing and Reporting the Results

**Figure 1:**
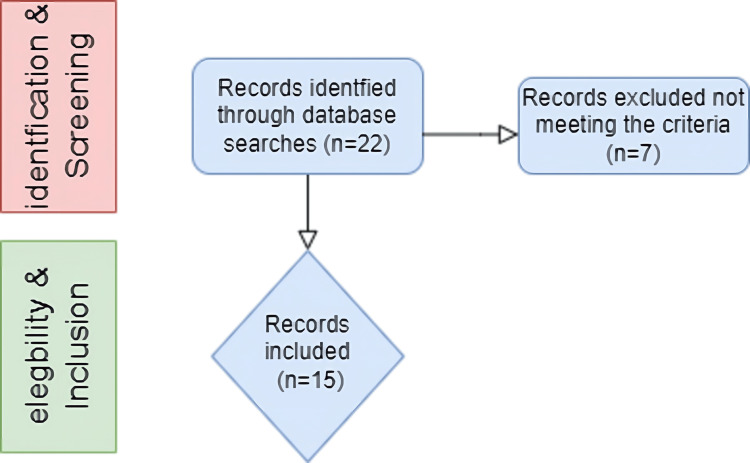
A thematic analysis of represented data.

Adhering to the scoping review methodology, we adopted an approach to summarize and report the data by our four research questions in a thematic analysis. We provided a narrative synthesis of the findings to help guide future research (Figure 1).

Results

We included 15 studies. The median number of patients included in the studies was 517.5 patients [3-6,9-19] (Table 1).

**Table 1:** Table illustrating the acquired and analyzed data.

Article	Number of participants	Type of surgery	U/S guided	Pain intraoperatively (opoid use)	PONV	Postoperative opoid use	Postoperative pain scale	Pain inward	Type of local	Concentration	Volume	Number of anesthesiologists	Catheter use
Hong et al. [3]	1,069	Radical mastectomy	Not mentioned	Lower in the PECS group	Not mentioned	Lower with PECS block	Lower with PECS block	Lower with PECS block	Not mentioned	Not mentioned	Not mentioned	Not mentioned	Not mentioned
Uribe et al. [4]	228	Simple mastectomy	Not mentioned	No difference	Increased with PECS block	No difference	No difference	Not mentioned	Not mentioned	Not mentioned	Not mentioned	Not mentioned	Not mentioned
Jin et al. [5]	1,120	Unspecified breast surgeries	Yes	Not mentioned	Lower in PECS block	Lower with PECS block	Lower with PECS block	Not mentioned	Not mentioned	Not mentioned	Not mentioned	Not mentioned	Not mentioned
Desroches et al, [6]	14	Unspecified breast surgeries	Yes	No difference	Not mentioned	More with PECS block	More with PECS	Not mentioned	Bupivacaine	0.25%	20 mL	Not mentioned	Not mentioned
Al Ja'bari et al. [9]	42	Radical mastectomy	Yes	No difference	Lower in PECS block	Lower with PECS block	Lower with PECS block	Not mentioned	Ropivacaine	0.50%	20 mL	Not mentioned	Not mentioned
Versyck et al. [10]	815	Unspecified breast surgeries	Yes	Lower in the PECS group	No difference	Lower with PECS block	Lower with PECS block	Not mentioned	Not mentioned	Not mentioned	Not mentioned	Not mentioned	Not mentioned
Senapathi et al. [11]	50	Radical mastectomy	Yes	Lower in the PECS group	Not mentioned	Lower with PECS block	Lower with PECS block	Not mentioned	Bupivacaine	0.25%	30 mL	Not mentioned	Not mentioned
Kim et al. [12]	80	Radical mastectomy	Yes	Lower in the PECS group	Not mentioned	Lower with PECS block	Lower with PECS block	Lower with PECS block	Ropivacaine	0.25%	30 mL	Not mentioned	Not mentioned
Kubodera et al. [13]	33	Radical mastectomy	Yes	Not mentioned	Not mentioned	Lower with PECS block	Lower with PECS block	Lower with PECS block	Ropivacaine	0.50%	30 mL	Not mentioned	Not mentioned
Bakeer et al. [14]	60	Radical mastectomy	Yes	No difference	Not mentioned	Lower with PECS block	Lower with PECS block	Lower with PECS block	Bupivacaine	0.25%	30 mL	Not mentioned	Not mentioned
de Cassai et al. [15]	140	Simple mastectomy	Yes	Lower in the PECS group	Not mentioned	Lower with PECS block	Lower with PECS block	Lower with PECS block	Levobupivacaine	0.25%	30 mL	Not mentioned	Not mentioned
Zhao et al. [16]	994	Radical mastectomy	Not mentioned	Lower in the PECS group	Lower in PECS block	Lower with PECS block	Lower with PECS block	No difference	Not mentioned	Not mentioned	Not mentioned	Not mentioned	Not mentioned
Bashandy et al. [17]	120	Radical mastectomy	Yes	Lower in the PECS group	Lower in PECS block	Lower with PECS block	Lower with PECS block	Lower with PECS block	Bupivacaine	0.25%	30 mL	Not mentioned	Not mentioned
Fancellu et al. [18]	207	Radical mastectomy	Yes	Lower in the PECS group	Lower in PECS block	Lower with PECS block	Lower with PECS block	No difference	Ropivacaine	0.38%	Not mentioned	Not mentioned	Not mentioned
Kurien et al. [19]	60	Radical mastectomy	Yes	Lower in the PECS group	Lower in PECS block	Lower with PECS block	Lower with PECS block	Lower with PECS block	Levobupivacaine	0.25%	30 mL	Not mentioned	Not mentioned

What Are the Uses of the PECS Block Regional Technique?

All studies mentioned above focused on the PECS block regional technique. The surgical procedures described in these studies varied with 60% (N = 9) that focused on radical mastectomy with and without lymph node dissection [3,9,11-14,16,17,19]. Furthermore, 26.6% (N = 4) were unspecified breast surgeries [5,10,15,18], and 13.3% (N = 2) focused on simple mastectomy [4,6].

Is the PECS Block Regional Technique Effective for Intraoperative and Postoperative Analgesia?

Intraoperative opioid use in the intervention groups showed a decrease in opioid consumption in 60% of the included articles (N = 9) [3,10-12,15-19], 27.7% (N = 4) showed no difference^ ^[4,6,9,14], and 13.3% (N = 2) did not mention any data about intraoperative opioid use [5,13].

Postoperative opioid use in the intervention groups showed a decrease in opioid consumption in the first 24 hours in 93.3% of the included articles (N = 14) [3,5,6,9,10-19], and 6.6% (N = 1) showed no difference in postoperative opioid consumption [4].

Postoperative pain levels in the intervention groups showed a decrease in patient-reported pain scores in 86.6% of the included articles (N = 13) [3,5,9-19], whereas 6.6% (N = 1) showed an increase in reported pain scores [6] and 6.6% (N = 1) showed no difference [4].

What Type of Local Anesthetics Was Used?

Of the 15 studies, 26.6% (N = 4) used ropivacaine [9,12,13,18], 26.6% (N = 4) used bupivacaine [6,11,14,17], 13.3% (N = 2) used levobupivacaine [15,19], and 3.3% (N = 5) did not mention the type of local anesthetic administered [3,5,10,16].

Discussion

This scoping review explores the current evidence of using the PECS regional block technique over the past years for chest wall surgeries. Facial plane blocks may have a role in improving intraoperative and postoperative pain relief and opioid consumption, quality of recovery, and patient satisfaction. The type, concentration, and volume of local anesthetic that can provide the optimum effect is yet to be determined for the PECS regional block.

Type of Surgery and Intraoperative Opioid Consumption

The reviewed patients underwent various breast surgeries, including unilateral modified mastectomy with or without sentinel lymph node dissection, breast augmentation, and breast-conserving surgery with lymph node dissection. Intraoperative opioid consumption was the only outcome in common among all the included articles in this review. Most studies showed a decrease in opioid use intraoperatively in the intervention groups [3,5,10-12,15-19]. Conversely, some randomized controlled trials showed no statistically significant difference in opioid consumption [4,6,14]. One study used sufentanil for every case [9].

This data shows that using the PECS block regional technique in breast surgeries is feasible and may effectively reduce intraoperative pain. The discrepancy in the studies that showed no statistically significant difference in intraoperative opioid consumption could be attributed to either a failed block (by location, type, concentration, or volume of the injectate), a low study power, or both.

Postoperative Nausea and Vomiting

Five articles reported a statistically significant decrease in the incidence of postoperative nausea and vomiting in the intervention groups [5,16-19]. Some of the other studies reported an increase in the incidence of postoperative nausea and vomiting [4,9], one showed no difference [10], and many did not include this outcome [3,6,11,13-15].

Immediate Postoperative Pain and Opioid Consumption

Most studies showed a statistically significant decrease in postoperative opioid consumption and patient-reported pain scores [3,5,9-19]. One study showed no difference in postoperative opioid consumption [4], and another showed an increased opioid consumption [6].^ ^The authors of this article suspect that part of the discrepancy in Maxim Roy’s trial is due to the unusual method of administering local anesthetic with the block on one side and a sham block (0.9% NaCl) on the other side making each patient their own control group, on top of the low number of patients included in their study (N = 19).

The available data suggest that the PECS regional block technique for breast surgery can be an effective modality in decreasing postoperative opioid consumption and patient-reported pain scores. This would lead to increased patient satisfaction and comfort while decreasing the risk of high-dose-opioid side effects such as postoperative nausea and vomiting and respiratory depression.

Postoperative Pain and Opioid Consumption in the First 24 Hours

Many studies did not document the full 24-hour postoperative period; however, in studies that did, multiple showed a decrease in patient-reported pain scores and the need for analgesics in the intervention groups [3,9,12-15,17,19]. Others showed no statistical difference [16,18].

The authors interpret the results in favor of the PECS regional block in providing, at least, some pain relief in the immediate 24 hours.

Injectate and Technique

The local anesthetic type, concentration, and volume used differed in each paper. Four studies used ropivacaine as their choice of local anesthetic in different concentrations [9,12,13,18]. Two used ropivacaine in a concentration of 0.5% with a total of 30 mL distributed as 20 mL between the pectoralis minor and serratus anterior muscles and 10 mL between the pectoralis minor and major muscles at the level of the third rib [9,13]. Two other studies used ropivacaine at concentrations of 0.25% and 0.375%, with a total of 30 mL injected [12,18]. Bupivacaine 0.25% was the choice of local anesthetic in four studies [6,11,14,17]. Levobupivacaine 0.25% was used in one study with a 0.2-0.4 mg/kg dose [19].

**Figure 2:**
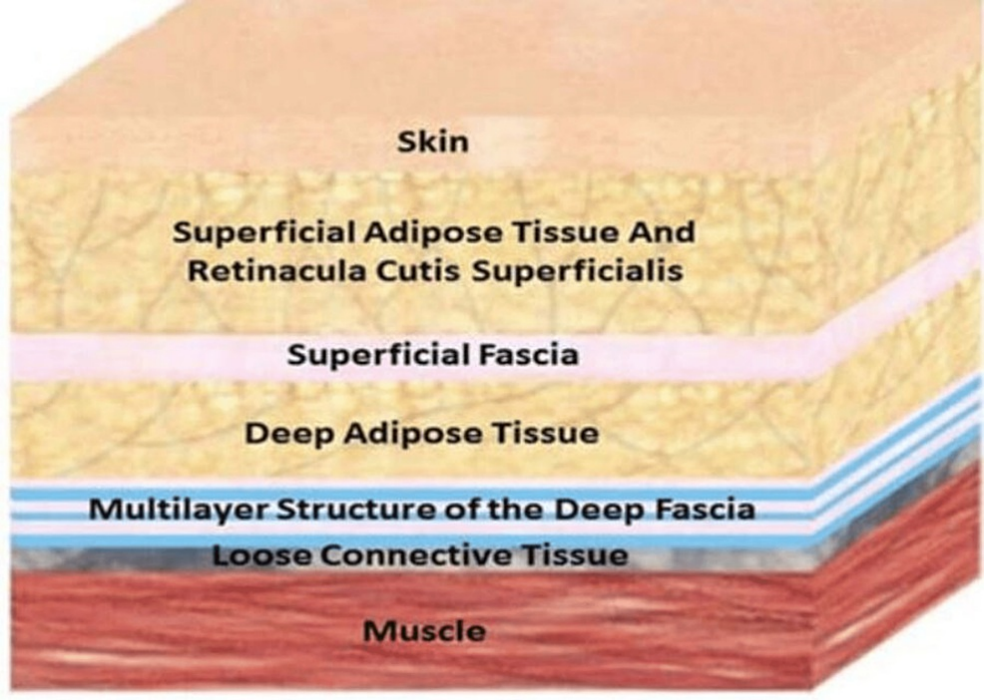
The two fascial connective layers: superficial fascia and deep fascia. Reproduced with permission from Elsharkawy et al. [20].

**Figure 3:**
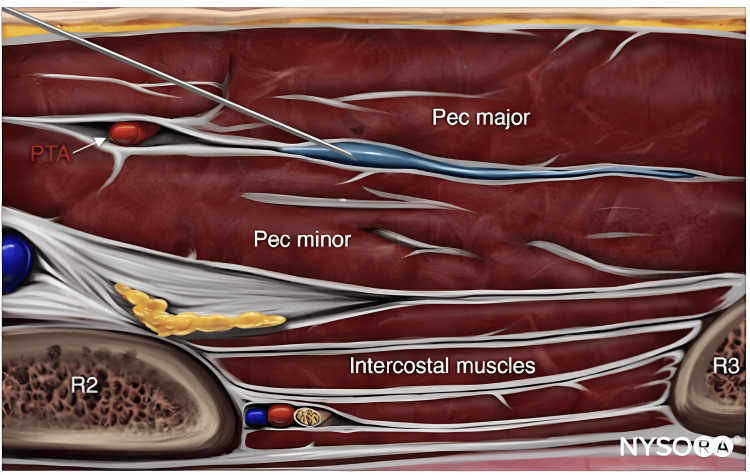
The anatomy of the PECS 1/2 block. Reproduced with permission from NYSORA.com [21].

All PECS regional blocks in the studies included in this scoping review were done preoperatively and with ultrasound guidance. However, this raises an important question and concern about the accuracy of the local anesthetic injection. Was it administered between the muscles? Or inside fascial layers? Or between the fascia, as described by et Elsharkawy et al. [20]. With multiple potential injection points, a clearer understanding of the anatomy is paramount for a successful block [22] (Figures 2, 3).

## Conclusions

Facial plane blocks may offer an innovative approach to the classical way of regional blocks. We target only the essential nerves to relieve intraoperative and postoperative pain and reduce opioid consumption. The persistent debate on their effectiveness can partly be explained by patients’ and providers’ observed high expectations and the underestimation of the needed provider skills in performing this seemingly easy but elusive block technique. The authors conclude that PECS block can provide a decrease in intraoperative and postoperative opioid consumption, a decrease in the incidence of nausea and vomiting, and can lead to overall patient satisfaction in terms of lower pain scores compared to systemic analgesia. The local anesthetic type, concentration, and volume vary widely between the studies, which paves the way for further research.
